# Recurrent SARS-CoV-2 RNA positivity and prolonged viral shedding in a patient with COVID-19: a case report

**DOI:** 10.1186/s12879-021-06776-3

**Published:** 2021-10-18

**Authors:** Chun-Hua Xiao, Lin-Fa Chen, You Li

**Affiliations:** 1 Department of Infectious Diseases, Huizhou Third People’s Hospital, Guangzhou Medical University, Huizhou, 516000 China; 2 Department of Neurology, Huizhou Third People’s Hospital, Guangzhou Medical University, Huizhou, 516000 China; 3 Guangdong Key Laboratory of Age-Related Cardiac and Cerebral Diseases, Affiliated Hospital of Guangdong Medical University, Zhanjiang, 524001 China

**Keywords:** Coronavirus, COVID-19, SARS-CoV-2, Recurrence, IgM, Viral shedding

## Abstract

**Background:**

The ongoing coronavirus disease 2019 (COVID-19) global pandemic caused by the SARS-CoV-2 virus remains a major threat to public health. At present, it is recommended that patients with known or suspected COVID-19 undergo quarantine or medical observation for 14 days. However, recurrent SARS-CoV-2 RNA positivity and prolonged viral shedding have been documented in convalescent COVID-19 patients, complicating efforts to control viral spread and ensure patient recovery.

**Case presentation:**

We report the case of a patient who experienced two recurrent episodes of SARS-CoV-2 RNA and IgM positivity and viral shedding over 60 days during hospitalization.

**Conclusions:**

This case report demonstrates that relapses of SARS-CoV-2 RNA and IgM positivity may occur even after COVID-19 symptoms have resolved, possibly as a consequence of prolonged viral shedding rather than re-infection.

**Supplementary Information:**

The online version contains supplementary material available at 10.1186/s12879-021-06776-3.

## Background

Coronavirus disease 2019 (COVID-19) is a pandemic disease that was first reported in Wuhan, China [1], and that has since spread to over 190 countries, causing over 46 million infections and 1.1 million deaths as of November 1st, 2020. Current World Health Organization guidelines indicate that COVID-19 patients can be discharged from the hospital when they are negative for SARS-CoV-2 viral RNA in two consecutive polymerase chain reaction (PCR) tests conducted ≥ 24 h apart [2]. However, there have been some reports of patients that exhibit prolonged viral RNA shedding even after they are no longer symptomatic [3]. There are also documented cases of SARS-CoV-2 recurrence in patients who have recovered from the disease [4, 5]. Herein, we report the case of a patient who experienced two episodes of recurrent SARS-CoV-2 RNA and IgM antibody positivity and viral RNA shedding over an 60-day period.

## Case presentation

**Fig. 1:**
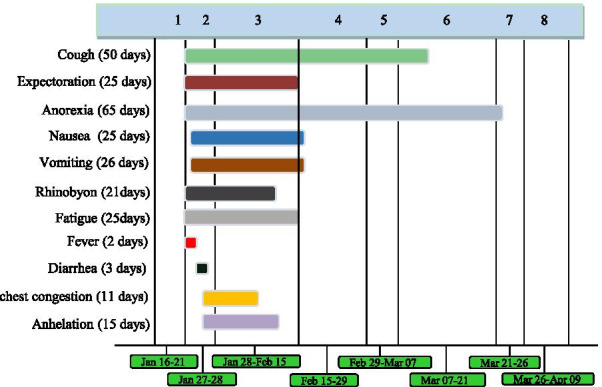
Symptoms according to day of illness, January 16 to April 09, 2020

**Fig. 2:**
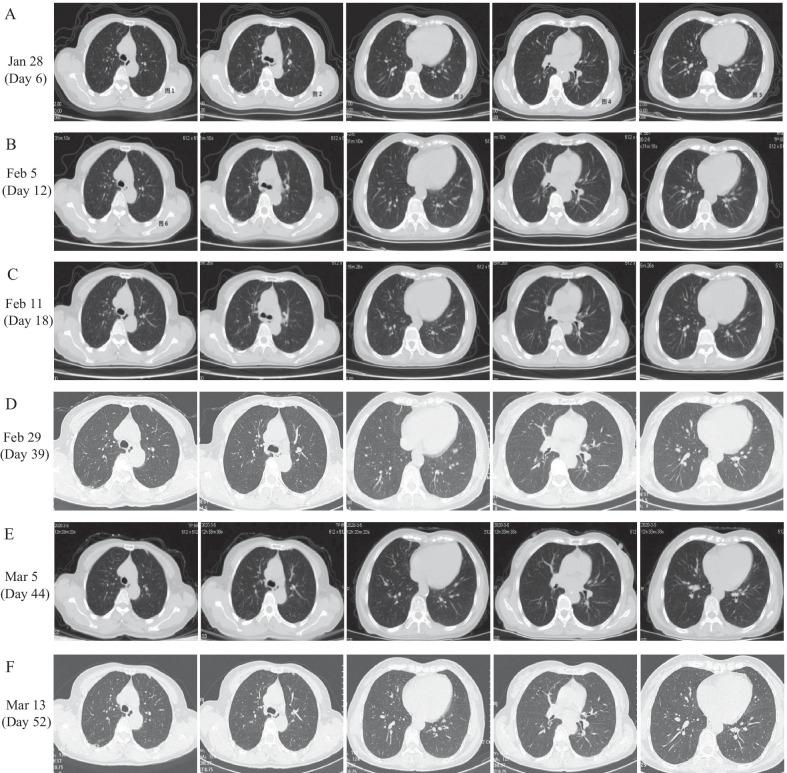
CT of the chest obtained on different time points of the disease course

**Fig. 3:**
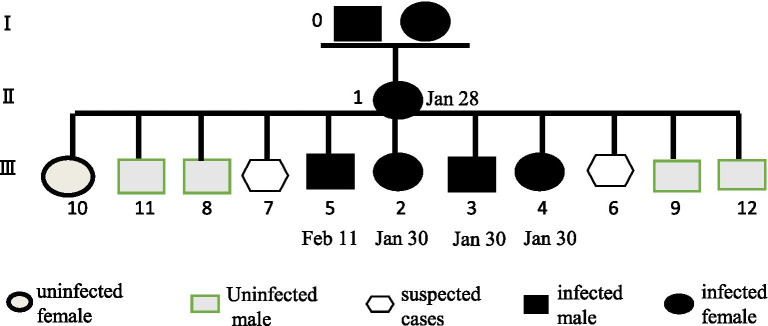
Schematic presentation of epdemiological investigation. 0, Unknown source of infection in Wuhan; 1, patient of the present study; 2, daughter-in-law of case 1; 3, older grandson of case 1; 4, daughter of case 1; 5, husband of case 1; 6, older sister of case 1; 7, younger sister of case 1; 8, son of case 1; 9, son-in-law of case 1; 10, granddaughter-in-law of case 1; 11, grandson-in-law of case 1; 12, younger grandson of case 1. The patient was diagnosed with COVID-19 on Jan 28. Her daughter-in-law was diagnosed with COVID-19 on Jan 30, while her husband was diagnosed with COVID-19 on Feb 11. Her daughter and older grandson were found to be asymptomatic carriers. Other family members were negative for SARS-CoV-2 infection at the time of testing

A 56-year-old woman who had traveled to Wuhan, China on January 2, 2020, presented with nasal congestion, cough, expectoration, anorexia, and fatigue on January 21, 2020. On January 22, she developed an intermittent fever lasting for 2 days, reaching a peak body temperature of 39.4 °C. She was treated with antibiotics (ceftriaxone) in a local clinic on January 23. She returned home to Huizhou on January 27, and reported to the fever clinic of Huizhou Third People’s Hospital on the following day with symptoms of nasal congestion, cough, expectoration, anorexia, nausea, vomiting, rhinorrhea, fatigue, fever, diarrhea, chest congestion, and dyspnea (Fig. 1). Initial blood tests revealed a slightly elevated lymphocyte count and normal oxygen saturation (Additional file 1: Table 1). Computed tomography (CT) scans showed multiple bilateral scattered regions of patchy ground-glass opacity in the lungs (Fig. 2A). RT-PCR tests (Additional file 2: Methods) of throat swabs collected later on the same day from this patient were positive for SARS-CoV-2 (ORF1 and N genes; cycle threshold values of approximately 22–23), and as such the patient was diagnosed with COVID-19 and transferred to an isolation ward. Her husband (Feb 11) and daughter-in-law (Jan 30) were also diagnosed with COVID-19, while her daughter (Jan 30) and older grandson (Jan 30) were found to be asymptomatic carriers (Fig. 3). Other family members were negative for SARS-CoV-2 infection at the time of testing (Fig. 3).

After hospitalization, the patient received moxifloxacin and arbidol for anti-infection therapy, methoxamine and ketotifen for anti-cough therapy, as well as pantoprazole for inhibition of gastric acid. Her symptoms improved significantly after symptomatic treatment (Fig. 2B, Additional file 1: Tables 1 and 2), but she was still positive for SARS-CoV-2 RNA on February 7 and 10 (Table 1). On February 11, routine blood tests, liver function tests, creatinine levels*,* and bilirubin levels were normal (Additional file 1: Tables 1 and 2), and chest CT scans indicated the resolution of the scattered bilateral lung lesions (Fig. 2C). On February 12 and 14, two consecutive oropharyngeal and anal swabs were found to be negative for SARS-CoV-2 RNA (Table 1). As per the recommendations of the New Coronavirus Infection Pneumonia Protocol (version 7) proposed by the National Health Commission of China, this patient was now eligible for discharge and release from isolation.

**Table 1:** Results of real-time reverse-transcriptase–polymerase-chain-reaction testing

Date/Specimen	Nasopharyngeal swab	Oropharyngeal swab	Anal swab	Blood sample
Jan 28 (illness day 7)	**Positive** (Ct, 22–23)	NT	NT	NT
Feb 07 (illness day 17)	**Positive** (N/A)	NT	NT	NT
Feb 10 (illness day 20)	**Positive** (N/A)	NT	NT	NT
Feb 12 (illness day 22)	NT	Negative	Negative	NT
Feb 14 (illness day 24)	NT	Negative	Negative	NT
Feb 29 (illness day 39)	NT	Negative	**Positive** (Ct, 36–37)	NT
Mar 03 (illness day 42)	NT	Negative	Negative	NT
Mar 04 (illness day 43)	NT	Negative	Negative	NT
Mar 06 (illness day 45)	NT	Negative	Negative	NT
Mar 14 (illness day 53)	NT	Negative	Negative	NT
Mar 21 (illness day 60)	NT	**Positive** (Ct, 36–36)	Negative	NT
Mar 25 (illness day 64)	NT	Negative	NT	NT
Mar 28 (illness day 67)	NT	NT	NT	Negative
Apr 02 (illness day 72)	NT	Negative	Negative	NT

A cycle threshold (Ct) value of 40 is ued as the cut-off point. Lower Ct values indicate higher viral loads

NT denotes “not tested”. N/A denotes “not available”

Positive nucleic acid test results are shown in bold

Given that SARS-CoV-2 viral clearance patterns remain poorly understood, all discharged COVID-19 patients are transferred to a designated clinical observation center for an additional 14-day quarantine. On February 15, this patient was still experiencing a cough and some sputum production (Fig. 1), although her other symptoms (anorexia, nausea, rhinorrhea, and fatigue) had improved. On February 28, she developed worsening anorexia and abdominal discomfort. On February 29, biochemical tests revealed slightly abnormal liver function (ALT40 U/L) (Additional file 1: Table 2), and a subsequent chest CT revealed the presence of a small number of bilateral fibrous lesions as well as the slight thickening of the left pleura (Fig. 2D). Anal swabs obtained on this same day were positive for SARS-CoV-2 RNA (cycle threshold values of approximately 36–37) (Table 1). The patient was thus transferred back to an isolation ward for further treatment. On March 5, chest CT follow-up demonstrated the resolution of the bilateral pulmonary lesions in this patient (Fig. 2E), and both throat and anal swab samples were found to be negative for SARS-CoV-2 RNA on March 3, March 4, and March 6 (Table 1). The patient was then discharged to a designated clinical observation center for an additional 14-day quarantine period.

On March 7, the patient experienced a nonproductive cough and anorexia (Fig. 1). A chest CT scan revealed the presence of a few fibrous bilateral pulmonary lesions on March 13 (Fig. 2F). On March 21, anal swab samples from this patient were found to be negative for SARS-CoV-2 RNA, whereas nasal swab samples were positive for viral RNA (cycle threshold values of approximately 36) (Table 1). IgM and IgG antibody tests (Additional file 2: Methods) specific for SARS-CoV-2 were positive on this same date (Additional file 1: Table 3). To minimize the risk of further viral transmission, the patient was admitted to the isolation ward for the third time. She was treated with oseltamivir. Oropharyngeal swab and blood samples tested negative for SARS-CoV-2 RNA on March 25 and March 28 (Table 1). The patient was discharged on Mach 28 and was transferred to the designated clinical observation center for an additional 14-day quarantine. IgM and IgG antibody tests specific for SARS-CoV-2 were positive on March 30 (Additional file 1: Table 3). Anal and nasal swabs from this patient were negative for SARS-CoV-2 RNA on April 2 (Table 1).

## Discussion and conclusion

There have been several reports to date of recurrent SARS-CoV-2 RNA positivity in patients who have recovered from COVID-19 [4–6]. In the present case, our patient had two consecutive negative RT-PCR tests of nasopharyngeal and oropharyngeal swabs after recovering from acute COVID-19 symptoms, but was subsequently found to exhibit SARS-CoV-2-positive anal and nasopharyngeal swabs on days 39 and 60 after symptom onset, respectively. Recently, Osman et al. performed a review of similar re-positive cases after discharge from hospitals in China, and concluded that such repositivization may be attributable to false-negative laboratory results and prolonged viral shedding, rather than to re-infection [7]. Although no official guidelines have been released to differentiate between whether recurrent RT-PCR positivity is a consequence of true COVID-19 recurrence or intermittent RNA shedding, a growing body of evidence supports that the viral RNA re-positivity is more likely to be the result of prolonged viral shedding rather than re-infection. Several studies have reported prolonged RT-PCR positivity in a significant proportion of recovered patients, however, RT-PCR re-positivity after recovery does not imply the presence of live or transmissible virus [8–10]. Viral culture tests failed to detect any virus growth in samples taken at Ct > 24 or for the first 8–18 days after symptom onset [11]. Recent animal model research further suggests that recovered rhesus macaques are resistant to SARS-CoV-2 re-infection [12]. Alexandersen et al. confirmed the presence of highly stable virus genomic and subgenomic RNAs in diagnostic swab samples, which may shed light on prolonged and sometimes inconsistent PCR-positivity [13]. Together, these lines of evidence thus suggest that the recurrence of SARS-CoV-2-RNA positivity may not be indicative of true re-infection.

Four members of this patient’s family who had been in close contact with her tested positive for SARS-CoV-2 RNA. The basic reproduction rate (R0) for SARS-CoV-2 in the present case was 5, consistent with the higher transmissibility of this virus relative to that of SARS-CoV (*R*0: 2–5) [14]. Further adaptation of SARS-CoV-2 to human hosts may further increase this R0 value, further expediting the global spread of this virus.

SARS-CoV-2 can be transmitted through both respiratory and extra-respiratory routes, which led us to recognize the importance of testing specimens from multiple sites to improve overall sensitivity and to reduce rates of false-negative test results. Nasopharyngeal swabs and/or oropharyngeal swabs are often recommended for patient screening or for the diagnosis of early-stage COVID-19 infections [15]. A single nasopharyngeal swab has become the standard sampling modality of choice, as it provides higher diagnostic yields, better patient tolerance, and is safer for operators. Nasopharyngeal swabs may also be combined with oropharyngeal swabs to increase sensitivity in the detection of SARS-CoV-2. Wang et al. were able to detect SARS-CoV-2 viral RNA in different types of clinical specimens, and found that bronchoalveolar lavage fluid samples exhibited the highest positivity rate, followed by sputum, nasopharyngeal swabs, fiberoptic bronchoscopy brush biopsy, oropharyngeal swabs, feces, and blood samples [16]. In order to improve the sensitivity of our detection assays in the present study, we analyzed at least two specimens from different sites, as viral shedding patterns may vary during different stages of infection. As anal swabs from this patient were positive for viral RNA after nasopharyngeal tests were negative, there is a possibility that viral shedding from the gastrointestinal tract may occur, potentially facilitating the fecal–oral transmission of SARS-CoV-2.

There are prior reports of prolonged SARS-CoV-2 shedding by COVID-19 patients even after symptoms have resolved and virus-specific antibodies have been detected. The patient in the present case report exhibited an 84-day clinical course, which was longer than the 37-day course reported in another recent study [17]. She had a cough that lasted for 56 days, as well as nausea and anorexia that lasted for 65 days, suggesting that SARS-CoV-2 was attacking both her respiratory and the digestive systems. This conclusion is further supported by the fact that both nasopharyngeal and anal swabs were positive for SARS-CoV-2 RNA. Even after the apparent resolution of lung pathology, this patient experienced cough, nausea, and anorexia, and recurrent SARS-CoV-2 RNA positivity. This indicates that viral shedding persisted in the respiratory and digestive tracts even after lung lesions had improved. Antibody tests may represent a valuable supplement to RT-PCR analyses when monitoring the course of COVID-19 disease in affected patients. Unexpectedly, the present case revealed that positive IgM antibodies were detected on days 60 after symptom onset. Li et al. recently reported that positive IgM and IgG titers were detected in 8 out of 16 discharged COVID-19 patients, suggesting the presence of active immunity and ongoing infection [18]. Qiu et al. [19] also found that IgM and IgG titers remained positive at day 51 after symptom onset, even when RT-PCR tests were negative. We thus speculate that the prolonged IgM positivity in our patient may be attributable to prolonged low-level viral shedding. During hospitalization, the patient received multiple antiviral drugs, suggesting that the duration of viral shedding may be prolonged despite such treatment [20]. Previous reports have suggested that the duration of viral shedding is correlated with the host’s immune status, as immunocompromised patients can shed influenza A virus for 18 months [21]. Many COVID-19 patients have low CD4^+^ and CD8^+^ T cell counts [21, 22]. Consistent with this, we observed low CD4^+^, CD8^+^, and CD3^+^ counts in the present patient on day 60 (Additional file 1: Table 4), at which time her nasopharyngeal swabs were again positive for SARS-CoV-2 viral RNA. We therefore speculate that the recurrent SARS-CoV-2 positivity in this patient may have been associated with impaired immune function, although further samples will need to be analyzed to confirm this hypothesis.

The limitations of this study should also be noted. First, we could not perform whole genome sequencing and viral culturing to confirm the RT-PCR results. Second, we did not titrate IgM and IgG and their ratios to determine whether there was sustained immune system maturation. Third, we did not monitor the viral load of SARS-CoV-2 at different stages of infection to verify nucleric acid results and viral shedding patterns.

In summary, the COVID-19 patient in this case report experienced two episodes of recurrent SARS-CoV-2 RNA positivity and prolonged viral shedding after the resolution of her symptoms, and was additionally positive for SARS-CoV-2-specific IgM at the time of recurrent shedding. This case emphasizes that it is important for discharged patients to comply with strict post-convalescent home isolation guidelines for at least 2 weeks, as SARS-CoV-2 RNA re-positivity or prolonged viral shedding may occur in some a subset of recovering patients. Further research is necessary in order to understand the mechanisms whereby SARS-CoV-2 RNA positivity recurs and viral shedding is prolonged in convalescent COVID-19 patients.

## Supplementary Information

**Additional file 1: Table 1.** Clinical routine blood tests. **Table 2.** Biochemical tests. **Table 3.** IgM and IgG antibodies against SARS-CoV-2. **Table 4.** Lymphocyte counts.

**Additional file 2: Methods**.

## Data Availability

All the data regarding the findings are available within the manuscript.

## Abbreviations

SARS-CoV-2: Severe acute respiratory syndrome coronavirus 2
COVID-19: Coronavirus disease 2019
Ct: Cycle threshold
CT: Computed tomography
R0: Reproduction rate

## References

[1] Hui DS, Azhar EI, Madani TA, Ntoumi F, Kock R, Dar O, Ippolito G, McHugh TD, Memish ZA, Drosten C (2020). The continuing 2019-nCoV epidemic threat of novel coronaviruses to global health—the latest 2019 novel coronavirus outbreak in Wuhan, China. Int J Infect Dis.

[2] Hoang VT, Dao TL, Gautret P (2020). Recurrence of positive SARS-CoV-2 in patients recovered from COVID-19. J Med Virol.

[3] Li N, Wang X, Lv T (2020). Prolonged SARS-CoV-2 RNA shedding: not a rare phenomenon. J Med Virol.

[4] Xiao AT, Tong YX, Zhang S (2020). False-negative of RT-PCR and prolonged nucleic acid conversion in COVID-19: rather than recurrence. J Med Virol.

[5] Yuan J, Kou S, Liang Y, Zeng J, Pan Y, Liu L (2020). PCR assays turned positive in 25 discharged COVID-19 patients. Clin Infect Dis.

[6] Lan L, Xu D, Ye G, Xia C, Wang S, Li Y, Xu H (2020). Positive RT-PCR test results in patients recovered from COVID-19. JAMA.

[7] Osman AA, Al Daajani MM, Alsahafi AJ (2020). Re-positive coronavirus disease 2019 PCR test: could it be a reinfection?. New Microbes New Infect.

[8] Cento V, Colagrossi L, Nava A, Lamberti A, Senatore S, Travi G, Rossotti R, Vecchi M, Casati O, Matarazzo E (2020). Persistent positivity and fluctuations of SARS-CoV-2 RNA in clinically-recovered COVID-19 patients. J Infect.

[9] Ren X, Ren X, Lou J, Wang Y, Huang Q, Shi Y, Deng Y, Li X, Lu L, Yan S (2021). A systematic review and meta-analysis of discharged COVID-19 patients retesting positive for RT-PCR. EClinicalMedicine.

[10] Azam M, Sulistiana R, Ratnawati M, Fibriana AI, Bahrudin U, Widyaningrum D, Aljunid SM (2020). Recurrent SARS-CoV-2 RNA positivity after COVID-19: a systematic review and meta-analysis. Sci Rep.

[11] Bullard J, Dust K, Funk D, Strong JE, Alexander D, Garnett L, Boodman C, Bello A, Hedley A, Schiffman Z (2020). Predicting infectious severe acute respiratory syndrome coronavirus 2 from diagnostic samples. Clin Infect Dis.

[12] Chandrashekar A, Liu J, Martinot AJ, McMahan K, Mercado NB, Peter L, Tostanoski LH, Yu J, Maliga Z, Nekorchuk M (2020). SARS-CoV-2 infection protects against rechallenge in rhesus macaques. Science.

[13] Alexandersen S, Chamings A, Bhatta TR (2020). SARS-CoV-2 genomic and subgenomic RNAs in diagnostic samples are not an indicator of active replication. Nat Commun.

[14] Chen J (2020). Pathogenicity and transmissibility of 2019-nCoV-A quick overview and comparison with other emerging viruses. Microbes Infect.

[15] Zou L, Ruan F, Huang M, Liang L, Huang H, Hong Z, Yu J, Kang M, Song Y, Xia J (2020). SARS-CoV-2 viral load in upper respiratory specimens of infected patients. N Engl J Med.

[16] Xing YH, Ni W, Wu Q, Li WJ, Li GJ, Wang WD, Tong JN, Song XF, Wing-Kin Wong G, Xing QS (2020). Prolonged viral shedding in feces of pediatric patients with coronavirus disease 2019. J Microbiol Immunol Infect.

[17] Zhou F, Yu T, Du R, Fan G, Liu Y, Liu Z, Xiang J, Wang Y, Song B, Gu X (2020). Clinical course and risk factors for mortality of adult inpatients with COVID-19 in Wuhan, China: a retrospective cohort study. Lancet.

[18] Li J, Wei X, Tian W, Zou J, Wang Y, Xue W, Xiao Q, Huang W (2020). Clinical features of discharged COVID-19 patients with an extended SARS-CoV-2 RNA positive signal in respiratory samples. Virus Res.

[19] Qiu X, Xiang Y, Sun J, Wang X, Chen G, Xu X, Liao S, Yang N, Li S, Yang G (2020). Dynamic changes of throat swabs RNA and serum antibodies for SARS-CoV-2 and their diagnostic performances in patients with COVID-19. Emerg Microbes Infect.

[20] Yan D, Liu XY, Zhu YN, Huang L, Dan BT, Zhang GJ, Gao YH (2020). Factors associated with prolonged viral shedding and impact of lopinavir/ritonavir treatment in hospitalised non-critically ill patients with SARS-CoV-2 infection. Eur Respir J.

[21] Weinstock DM, Gubareva LV, Zuccotti G (2003). Prolonged shedding of multidrug-resistant influenza A virus in an immunocompromised patient. N Engl J Med.

[22] Hao S, Lian J, Lu Y, Jia H, Hu J, Yu G, Wang X, Xu K, Ni Q, Li Y (2020). Decreased B cells on admission was associated with prolonged viral RNA shedding from respiratory tract in coronavirus disease 2019: a case control study. J Infect Dis.

