# Supplementary material for: Recurrent SARS-CoV-2 RNA positivity and prolonged viral shedding in a patient with COVID-19: a case report
Source: BMC Infect Dis. 2021 Oct 18;21:1076. doi: 10.1186/s12879-021-06776-3 (PMC8523010; doi:10.1186/s12879-021-06776-3)
Supplement: Supplementary file 1 — Additional file 1: Table 1. Clinical routine blood tests. Table 2. Biochemical tests. Table 3. IgM and IgG antibodies against SARS-CoV-2. Table 4. Lymphocyte counts. [file 12879_2021_6776_MOESM1_ESM.docx]

**Additional Table 1 Clinical routine blood tests.**

| Blood Routine Test | Jan 28  (Day 7 ) | Jan 30  (Day 9 ) | Feb 05  (Day 15 ) | Feb 11  (Day 21 ) | Feb 29  (Day 39 ) | Mar 03  (Day 42 ) | Mar 09  (Day 48) | Mar 14  (Day 53) | Mar 21  (Day 60 ) | Mar 22  (Day 61 ) | Mar 28  (Day 67 ) | Mar 30  (Day 69 ) | Apr 02  (Day 72) | Reference  range |
| --- | --- | --- | --- | --- | --- | --- | --- | --- | --- | --- | --- | --- | --- | --- |
| WBC (10^9^/L) | 6 | 5.68 | 4.22 | 5.34 | 5.6 | 5.41 | 4.6 | 6.5 | 7.1 | 5.26 | 6.8 | 5.7 | 5.7 | 3.5-9.5 |
| Lym (10^9^/L) | 1.28 | 1.34 | 1.14 | 1.33 | 1.4 | 1.2 | 1.4 | 1.1 | 2.2 | 1.67 | 1.6 | 1.3 | 1.3 | 1.10-3.20 |
| Mon (10^9^/L) | **0.68** | 0.51 | 0.42 | 0.46 | 0.4 | 0.46 | 0.3 | 0.4 | 0.5 | 0.34 | 0.5 | 0.4 | 0.4 | 0.10-0.60 |
| NEUT (10^9^/L) | 4.02 | 3.79 | 2.8 | 3.49 | 3.8 | 3.67 | 2.9 | 4.8 | 4.3 | 3.18 | 4.6 | 3.9 | 4 | 1.80-6.30 |
| RBC (10^12^/L) | 4.59 | 4.77 | 4.74 | 4.61 | 4.45 | 4.22 | 4.36 | 4.55 | 4.85 | 4.58 | 4.8 | 4.65 | 4.8 | 3.80-5.10 |
| HCT (%) | 38.7 | 38.8 | 38.1 | 37.4 | 38.4 | 35 | 37.1 | 39 | 41.3 | 40.8 | 40.9 | 39.1 | 40.2 | 35.0-45.0 |
| HGB (g/L) | 133 | 138 | 135 | 133 | 129 | 122 | 127 | 133 | 140 | 136 | 138 | 134 | 138 | 115-150 |
| PLT (10^9^/L) | 195 | 267 | 293 | 254 | 249 | 271 | 247 | 232 | 258 | 245 | 230 | 196 | 244 | 125-350 |
| MPV (fl) | 9.1 | 9.6 | 10.1 | 10.5 | 9.2 | 10 | 8.8 | 8.8 | 8.5 | 8.3 | 9 | 9.8 | 8.9 | 6.0-11.0 |
| PCT (%) | 0.18 | 0.26 | 0.3 | 0.27 | 0.2 | 0.27 | 0.2 | 0.2 | 0.2 | 0.2 | 0.2 | 0.2 | 0.2 | 0.1-0.5 |
| PDW (%) | 8.7 | 10.2 | 11.4 | 13.3 | 9.3 | 11.3 | 8.9 | 8.9 | 8.6 | 15.8 | 9 | 19 | 38 | 9.0-17 |

Abnormal values were in bold font.

WBC, white blood cell count; Lym, lymphocyte count; Mono, monocyte; NEUT, [neutrophil](http://www.youdao.com/w/neutrophil/#keyfrom=E2Ctranslation); RBC, red blood cell; HCT, hematocrit; HGB, [hemoglobin](http://www.youdao.com/w/hemoglobin/#keyfrom=E2Ctranslation); PLT, platelet; MPV, [mean platelet volume](http://www.youdao.com/w/mean%20platelet%20volume/#keyfrom=E2Ctranslation); PCT, [thrombocytocrit](http://www.youdao.com/w/thrombocytocrit/#keyfrom=E2Ctranslation); PDW, [platelet distribution width](http://www.youdao.com/w/platelet%20distribution%20width/#keyfrom=E2Ctranslation)

**Additional Table 2 Biochemical tests.**

|  | Jan 30  (Day 9) | Feb 05  (Day 15) | Feb 11  (Day 21) | Feb 29  (Day 39) | Mar 03  (Day 42) | Mar 09  (Day 48) | Mar 22  (Day 61) | Mar 28  (Day 67) | Reference range |
| --- | --- | --- | --- | --- | --- | --- | --- | --- | --- |
| ALT (U/L) | 17 | 16 | 18 | **40** | **83** | 32 | **79** | 33 | 5-35 |
| AST (U/L) | 34 | 27 | 31 | 35 | **88** | 23 | **74** | 27 | 8-40 |
| GGT (U/L) | 15 | 13 | 11 | 10 | 12 | 10 | 14 | 11 | 7-45 |
| ALP (U/L) | 46 | 53 | 62 | 97 | 51 | 89 | 76 | 103 | 50-135 |
| K (mmol/L) | **3.19** | 4.01 | 4.01 | **3.39** | 3.64 | 3.65 | **3.43** | / | 3.5-5.3 |
| Na (mmol/L) | **133.1** | 139.7 | 139.3 | 138 | 140.2 | 138 | 142.2 | / | 137-147 |
| Cl (mmol/L) | **92.2** | 102.1 | 101.9 | 103.6 | 99.2 | 102.9 | 103.32 | / | 99-110 |
| Ca (mmol/L) | 2.21 | 2.33 | 2.37 | 2.41 | 2.23 | 2.36 | 2.29 | / | 2.25-2.75 |
| TCO_2_ (mmol/L) | **32.8** | **33.5** | **34.4** | 24.63 | **31** | 26.34 | **30.33** | / | 22.0-29.0 |
| UREA (mmol/L) | 4.66 | 4.94 | 4.06 | 3.96 | 4.79 | 4.6 | 5.9 | / | 2.6-7.5 |
| CR (µmol/L) | **51.6** | **48.9** | **42.5** | **45** | **37.5** | **50.7** | **53.3** | / | 62.0-115.0 |
| GLU (mmol/L) | 5.25 | 5.81 | 4.89 | **7.38** | 4.44 | 5.77 | **3.78** | / | 3.89-6.11 |
| AGP | 8.1 | **4.1** | **3** | 11 | 10 | 10.1 | 8.6 | / | 8.0-16.0 |
| Osm (mosm/kg) | **266.8** | **279.9** | **277.9** | 294.1 | **279.3** | 293.7 | 283.6 | / | 280.0-320.0 |
| CK (U/L) | 45 | 45 | 45 | 40 | 47 | / | **28** | / | 40-200 |
| CKMB (U/L) | 7.9 | 3.6 | 5.1 | 18 | 1 | / | 7.3 | / | 0-25 |
| TP (g/L) | 67.9 | 66.5 | 70.8 | 72.4 | 68.5 | / | 77.3 | 77.5 | 65.0- 85.0 |
| ALB (g/L) | **33.5** | **32.8** | **36.1** | 45.9 | **35.3** | / | 42.8 | 42.8 | 40.0-55.0 |
| GLB (g/L) | 34.3 | 33.7 | 34.7 | 26.5 | 33.2 | / | 34.5 | 34.7 | 20.0-40.0 |
| TBIL (µmol/L) | 10.9 | 18.7 | 19.4 | 11.64 | **20.6** | / | 17.3 | 16.5 | 3.4-20.0 |

Abnormal values were in bold font.

ALT, [alanine aminotransferase](http://www.youdao.com/w/alanine%20aminotransferase/#keyfrom=E2Ctranslation); AST, [aspartate aminotransferase](http://www.youdao.com/w/aspartate%20aminotransferase/#keyfrom=E2Ctranslation); GGT,  gamma-glutamyltransferase; ALP, [alkaline phosphatase](http://www.youdao.com/w/alkaline%20%20phosphatase/#keyfrom=E2Ctranslation); K, potassium, Na, Sodium; Cl,  [chlorine](http://www.youdao.com/w/chlorine/#keyfrom=E2Ctranslation); Ca,  [calcium](http://www.youdao.com/w/calcium/#keyfrom=E2Ctranslation); TCO2, Total carbon dioxide; CR, [creatinine](http://www.youdao.com/w/creatinine/#keyfrom=E2Ctranslation); GLU, glucose; AGP, [anion gap](http://www.youdao.com/w/anion%20gap/#keyfrom=E2Ctranslation); Osm, [osmotic pressure](http://www.youdao.com/w/osmotic%20pressure/#keyfrom=E2Ctranslation); CK, [creatine kinase](http://www.youdao.com/w/CK(creatine%20kinase)/#keyfrom=E2Ctranslation); CKMB, [Creatine Kinase Isoenzyme](http://www.youdao.com/w/Creatine%20Kinase%20Isoenzyme/#keyfrom=E2Ctranslation); TP, total protein; ALB, [albumin](http://www.youdao.com/w/albumin/#keyfrom=E2Ctranslation); GLB,  [globulin](http://www.youdao.com/w/globulin/#keyfrom=E2Ctranslation); TBIL, total bilirubin

**Additional Table 3 IgM and IgG** antibodies against SARS-CoV-2

| Antibodies | Mar 21  (Day 60) | Mar 30  (Day 69) |
| --- | --- | --- |
| IgM | positive | positive |
| IgG | positive | positive |

**Additional Table 4 Lymphocyte counts**

| Lymphocyte | Mar 09  (Day 48) | Mar 14  (Day 53) | Mar 21  (Day 60) | Mar 30  (Day 69) | Reference |
| --- | --- | --- | --- | --- | --- |
| CD_4_ | 572 | 752 | 564 | 548 | 500-1440 |
| CD_8_ | 252 | 280 | 264 | **220** | 238-1250 |
| CD_3_ | 880 | 1084 | 868 | 808 | 770-2860 |
| CD_4_/CD_8_ | 2.27 | 2.69 | 2.14 | 2.49 | 1.0-2.47 |
