# Supplementary material for: Recurrent SARS-CoV-2 RNA positivity and prolonged viral shedding in a patient with COVID-19: a case report
Source: BMC Infect Dis. 2021 Oct 18;21:1076. doi: 10.1186/s12879-021-06776-3 (PMC8523010; doi:10.1186/s12879-021-06776-3)
Supplement: Supplementary file 2 — Additional file 2: Methods. [file 12879_2021_6776_MOESM2_ESM.docx]

**Methods**

As of Apr 2, 2020, COVID-19 patients of Huizhou city who met all of the hospital discharge criteria were requested to stay in medical isolation observation for a further 14 days, and the discharge criteria includes:

1. Body temperature below 37 degrees, lasting for at least three consecutive days;

2. Resolved respiratory symptoms;

3. Substantially improved chest lesions on computed tomography (CT) images; and

4. Two consecutive negative RT-PCR test results with at least a 1-day interval.

The clinical classification of COVID-19 is defined clearly in the “Diagnosis and Treatment of Pneumonia Caused by Novel Coronavirus (Trial Version 7)”. In brief, the mild type has no signs of pneumonia on chest imaging; the moderate type includes fever and respiratory symptoms, and signs of pneumonia on radiologic assessment; the severe type meets any of the following criteria: (1) shortness of breath, RR ≥ 30 times/min; (2) oxygensaturation ≤ 93% at rest; (3) arterial oxygen partial pressure/fraction of inspiration O_2_ (PaO_2_/FiO_2_ ≤ 300 mmHg); and (4) pulmonary imaging showing significant progression of lesion > 50% within 24–48 h.

**Data collection**

We reviewed clinical records, laboratory findings, and chest CT scans for the patient. Two study

investigators independently reviewed the data. Nasopharyngeal swab, Oropharyngeal swab, Anal

swab and blood samples were collected and tested for SARS-CoV-2, following WHO guidelines for qRT-PCR.

**The procedure of real-time RT-PCR Kit**
RT-PCR assays were performed using the real-time RT-PCR Kit (Shanghai ZJ Bio-Tech Co, Ltd,

Shanghai, China), targeting the open reading frame lab (ORF1ab) and nucleoprotein (N) gene

regions.The PCR parameters were 45 °C for 10 min, 95 °C for 3 min, followed by 45 cycles of 95 °C

for 15 s, 58 °C for 30 s, and a single fluorescence detection point at 58°C. When two targets tested

positive by specific real-time RT-PCR, the case would be considered to be positive. A cycle threshold

value (Ct-value) less than 37 was defined as a positive test, and a Ct-value of 40 or more was defined

as a negative test. A medium load, defined as a Ct-value of 37 to less than 40, required confirmation by

retesting. If the sample was tested positive using the kit provided by Shanghai ZJ Bio-Tech Co, Ltd,

Shanghai, China, it was retested using a real-time RT-PCR kit provided by Jiangsu Bioperfectus

Technologies Co., Ltd, Jiangsu, China. Only both were positive, case can be considered to be

laboratory-confirmed.

**Detection of anti-SARS-CoV-2 antibodies**

We detect IgM and IgG against SARS-CoV-2 using a SARS-CoV-2 Antibody test Strip (Sinocare, Changsha, China) based on Colloidal Gold method. The manufacturer reported a 99.5% sensitivity and specificity of 99.8% after 14 days post-PCR confirmation. This test uses a recombinant protein representing the nucleocapsid (N) antigen for the determination of antibodies against SARS‐CoV‐2 in the serum sample.
